# Effects of Autumn and Spring Heat Waves on Seed Germination of High Mountain Plants

**DOI:** 10.1371/journal.pone.0133626

**Published:** 2015-07-21

**Authors:** Simone Orsenigo, Thomas Abeli, Graziano Rossi, Paolo Bonasoni, Cristian Pasquaretta, Maurizia Gandini, Andrea Mondoni

**Affiliations:** 1 Department of Earth and Environmental Sciences, University of Pavia, Pavia, Italy; 2 Institute of Atmospheric Sciences and Climate, Bologna, Italy; 3 Institut Pluridisciplinaire Hubert Curien, CNRS, Strasbourg, France; 4 MUSE–Science Museum, Trento, Italy; US Geological Survey, UNITED STATES

## Abstract

Alpine plants are considered to be particularly vulnerable to climate change and related extreme episodes, such as heat waves. Despite growing interest in the impact of heat waves on alpine plants, knowledge about their effects on regeneration is still fragmentary. Recruitment from seeds will be crucial for the successful migration and survival of these species and will play a key role in their future adaptation to climate change. In this study, we assessed the impacts of heat waves on the seed germination of 53 high mountain plants from the Northern Apennines (Italy). The seeds were exposed to laboratory simulations of three seasonal temperature treatments, derived from real data recorded at a meteorological station near the species growing site, which included two heat wave episodes that occurred both in spring 2003 and in autumn 2011. Moreover, to consider the effect of increasing drought conditions related to heat waves, seed germination was also investigated under four different water potentials. In the absence of heat waves, seed germination mainly occurred in spring, after seeds had experienced autumn and winter seasons. However, heat waves resulted in a significant increase of spring germination in c. 30% of the species and elicited autumn germination in 50%. When heat waves were coupled with drought, seed germination decreased in all species, but did not stop completely. Our results suggest that in the future, heat waves will affect the germination phenology of alpine plants, especially conditionally dormant and strictly cold-adapted chorotypes, by shifting the emergence time from spring to autumn and by increasing the proportion of emerged seedlings. The detrimental effects of heat waves on recruitment success is less likely to be due to the inhibition of seed germination *per se*, but rather due to seedling survival in seasons, and temperature and water conditions that they are not used to experiencing. Changes in the proportion and timing of emergence suggest that there may be major implications for future plant population size and structure.

## Introduction

Climate warming is one of the main drivers of future ecosystem changes [1]. Current knowledge about plant response to climate change is largely based on the effects of climatic trends, such as gradual warming. However, over the last twenty years, extreme weather events, such as heat waves, drought, late frost events and heavy and irregular rainfalls have increased in frequency and intensity [2,3,4,5], bringing about changes to ecosystems [6,7]. In this regard, heat waves (defined by the WMO World Meteorological Organization as a period in which the daily maximum temperature of more than five consecutive days exceeds the average maximum temperature by 5°C, the normal period being 1961–1990) are one of the most studied phenomena [8,9], having shown a significant increase in frequency and duration in the northern hemisphere [10,11,12]. Heat waves are known to have negative effects on plants, especially when they are associated with intense drought [13,14,15,16].

Recent climate change has already had an impact on biological systems worldwide, and mountain ecosystems are considered particularly susceptible [17,18]. In response to gradual warming, phenological shifts have already been observed in alpine plants [19], with expansion or compression of the reproductive period [20], thermophilization of communities [21], alteration of species composition, or species migration and extinction [22,23,24]. However, the effect of climate extremes in these environments is still unclear, as they have been shown to have both positive and negative impacts on plants [25]. For instance, heat waves can either have a direct impact on the reproductive performance of arctic-alpine species by reducing the number of flowers [26], or an indirect impact through advanced snowmelt, causing subsequent frost damage [27]. Conversely, heat waves may favour plant performance (in the absence of drought), by increasing photosynthetic activity [28] and overall plant fitness through acclimation to warmer conditions [29].

In the future, to cope with temperature increase and extreme events, alpine species will need to adapt or migrate [30]. As both adaptation and migration depend on the capacity of plant populations to regenerate from seeds, successful seedling recruitment will play a key role for species survival in warmer climates [31]. Temperature and water availability are the most important environmental variables that control seed germination and seedling survival [32], so understanding their effects on recruitment success will help to highlight mechanisms of plant adaptation. Indeed, the effects of climate change on plant regeneration from seeds have received increasing attention [31], and studies have been performed in the laboratory [33,34,35], in greenhouses [36] and in the field [37,38,39]. Despite this attention, little is known about the effects of heat waves on the seed germination of alpine plants. However, extreme heating and water evaporation may prevent and/or delay seed germination, which may subsequently affect seedling survival and plant fitness, and, in turn, lead to changes in local species dominance in alpine plant communities.

The present study investigated the effects of short-term heat events on the germination of alpine plants by exposing the seeds of 53 high mountain species that grow in the Northern Apennines (Italy) to different temperature treatments and water potentials in the lab, simulating heat waves that occurred in southern Europe in spring 2003 and autumn 2011. Our main research goals were to determine: 1) whether heat waves enhance and/or shift seed germination, 2) how seed germination is affected when heat waves are coupled with drought, 3) whether germination responses differ across species and seasons, and whether these potential differences are related to species biogeographic distribution.

## Material and Methods

### Study species

The study was performed in the laboratory; however, seed collection was planned according to protected areas legislation. The Appennino Tosco-Emiliano National Park and Frignano Regional Park (N-Italy) issued the permission for seed collection. Seeds were collected according to the ENSCONET Manual protocol, i.e. no more than 20% of the total mature seeds available were collected (at least 700 seeds per species) and the natural plant populations were respected. The study did not involve endangered or protected species. Seeds were collected at the time of natural dispersal [40] in August 2012 from 53 species that are representative of the habitats and plant communities that grow above the tree-line of the Northern Apennines (Table 1) [41]. This mountain range is characterised by low altitude mountains (few summits exceed 2000 m a.s.l.) and a large number of boreal and alpine plant species at the edge of their southern geographical distribution [42]. In order to investigate whether different geographical distribution influenced species response to heat waves, we grouped all the species according to their chorotype (Table 1). Chorotypes are distribution categories of a group of organisms with similar geographical distribution, regardless of their biogeographic regionalisation [43]; they are based on an analysis of the distribution of species with the goal of determining patterns of distribution. A total of seven chorotypes were distinguished according to Alessandrini *et al*. [42]. To facilitate reading, each species will hereafter be referred to by its genus name, except for those belonging to the same genus, where the full name will be given. For each species, we used seeds from a single population, randomly collected from at least 100 individuals. The species were mainly collected at Mt. Cimone (44 19.3′N; 10 70.0′E; 2165 m a.s.l.), the highest peak of the Northern Apennines, and at the nearby peaks of Mt. Rondinaio (44 11.4’N; 10 59.7’E; 1964 m a.s.l.) and Mt. Giovo (44 13.1’N; 10 57.7’E; 1991 m a.s.l.). In order to include a wide representativeness of native flora, some species were also collected a few kilometers away in the areas of Mt. Libro Aperto (44 09.4’N; 10 42.7’E; 1937 m a.s.l.), Mt. Cusna (44 16.6’N; 10 24.5’E; 2070 m a.s.l.) and Mt. Prado (44 25.0’N; 10 40.7’E; 2054 m a.s.l.) (Table 1). All the peaks included in the study spanned an area of approximately 150 km^2^. Distances between seed collection sites and the automatic weather station ranged from a few meters to 27 km in Mt. Cusna. Immediately after harvesting the seeds, they were taken to laboratories at the University of Pavia, cleaned and sown on agar to test germination.

**Table 1:** List of species used in the experiment. Nomenclature follows Conti *et al*. 2005 [64] and Peruzzi *et al*. 2010 [65] and successive updates.

Family	Species	Chorotype	Collection site	Elevation (m a.s.l.)
*Amaryllidaceae*	***Allium schoenoprasum* L.**	Circumboreal	Mt. Rondinaio	1775
*Ranunculaceae*	***Anemonastrum narcissiflorum* (L.) Holub**	Circumboreal	Mt. Rondinaio	1775
*Asteraceae*	***Antennaria dioica* (L.) Gaertn.**	Circumboreal	Mt. Cimone	1895
*Poaceae*	***Anthoxanthum alpinum* Á.Löve & D.Löve**	Arctic-Alpine	Mt. Cimone	1875
*Plumbaginaceae*	***Armeria marginata* (Levier) Bianchini**	Endemic	Mt. Cimone	1950
*Asteraceae*	***Aster alpinus* L.**	Arctic-Alpine	Mt. Cimone	2110
*Poaceae*	***Brachypodium genuense* (DC.) Roem. & Schult.**	Endemic	Mt. Cimone	1875
*Asteraceae*	***Carduus defloratus* subsp. *carlinifolius* (Lam.) Ces.** (= *Carduus carlinifolius* Lam.)	Orophitic-European	Mt. Cimone	1875
*Cyperaceae*	***Carex foetida* All.**	Orophitic-European	Mt. Cusna	2050
*Asteraceae*	***Centaurea nervosa* Willd.**	Orophitic-European	Mt. Cimone	1875
*Asteraceae*	***Cirsium bertolonii* Spreng.**	Endemic	Mt. Cimone	1875
*Poaceae*	***Deschampsia cespitosa* (L.) P.Beauv subsp. *cespitosa***	Subcosmopolitan	Mt. Cimone	1875
*Caryophyllaceae*	***Dianthus deltoides* L.**	Eurasiatic	Mt. Cimone	1875
*Ericaceae*	***Empetrum hermaphroditum* Hagerup**	Circumboreal	Mt. Prado	1900
*Cyperaceae*	***Eriophorum angustifolium* Honck.**	Circumboreal	Mt. Rondinaio	1600
*Cyperaceae*	***Eriophorum latifolium* Hoppe**	Eurasiatic	Mt. Rondinaio	1600
*Poaceae*	***Festuca alfrediana* Foggi & Signorini**	Orophitic-European	Mt. Cimone	2030
*Poaceae*	***Festuca rubra* subsp. *commutata* (Gaudin) Markgr.-Dann.** (= *Festuca nigrescens* Lam.)	Circumboreal	Mt. Cimone	1875
*Poaceae*	***Patzkea paniculata* (L.) G.H.Loos** (= *Festuca paniculata* (L.) Schinz & Thell)	Orophitic-European	Mt. Giovo	1740
*Poaceae*	***Festuca riccerii* Foggi & Graz.Rossi**	Endemic	Mt. Cimone	1875
*Poaceae*	***Festuca violacea* Schleich. ex Gaudin subsp. *puccinelli* (Parl.) Foggi, Graz.Rossi & Signorini**	Endemic	Mt. Cimone	2030
*Fabaceae*	***Genista radiata* (L.) Scop.**	Orophitic-European	Mt. Cimone	1875
*Gentianaceae*	***Gentiana kochiana* E.P.Perrier & Songeon**	Orophitic-European	Mt. Cimone	1875
*Gentianaceae*	***Gentiana purpurea* L.**	Orophitic-European	Mt. Cimone	1960
*Geraniaceae*	***Geranium argenteum* L.**	Orophitic-European	Mt. Cimone	2110
*Rosaceae*	***Geum montanum* L.**	Orophitic-European	Mt. Cimone	1875
*Asteraceae*	***Gnaphalium supinum* L.**	Circumboreal	Mt. Rondinaio	1885
*Asteraceae*	***Homogyne alpina* (L.) Cass.**	Orophitic-European	Mt. Cimone	1875
*Hypericaceae*	***Hypericum richeri* Vill. subsp. *richeri***	Orophitic-European	Mt. Cimone	1960
*Juncaceae*	***Juncus alpinoarticulatus* Chaix subsp. *alpinoarticulatus***	Circumboreal	Mt. Cimone	1875
*Juncaceae*	***Juncus filiformis* L.**	Circumboreal	Mt. Cimone	1875
*Juncaceae*	***Juncus trifidus* L.**	Circumboreal	Mt. Libro Aperto	1925
*Juncaceae*	***Luzula alpinopilosa* (Chaix) Breistr. subsp. *alpinopilosa***	Circumboreal	Mt. Cimone	2110
*Juncaceae*	***Luzula lutea* (All.) DC.**	Orophitic-European	Mt. Cimone	1950
*Juncaceae*	***Luzula multiflora* (Ehrh.) Lej. subsp. *multiflora***	Circumboreal	Mt. Cimone	1875
*Juncaceae*	***Luzula spicata* (L.) DC. subsp. *spicata***	Orophitic-European	Mt. Cimone	1895
*Poaceae*	***Nardus stricta* L.**	Eurosiberian	Mt. Lagoni	1950
*Poaceae*	***Phleum alpinum* L.**	Orophitic-European	Mt. Cimone	1850
*Plantaginaceae*	***Plantago alpina* L. subsp. *alpina***	Orophitic-European	Mt. Cimone	1875
*Ranunculaceae*	***Pulsatilla alpina* (L.) Delarbre subsp. *millefoliata* (Bertol.) D.M. Moser**	Eurosiberian	Mt. Rondinaio	1885
*Polygonaceae*	***Rumex scutatus* L. subsp. *scutatus***	Eurosiberian	Mt. Cimone	1950
*Rosaceae*	***Sanguisorba officinalis* L.**	Circumboreal	Mt. Cimone	1875
*Saxifragaceae*	***Saxifraga exarata* Vill. subsp. *moschata* (Wulfen) Cavill.**	Orophitic-European	Mt. Cimone	2110
*Saxifragaceae*	***Saxifraga oppositifolia* L. subsp. *oppositifolia***	Circumboreal	Mt. Cimone	2110
*Saxifragaceae*	***Saxifraga paniculata* Mill.**	Circumboreal	Mt. Cimone	1950
*Caprifoliaceae*	***Scabiosa lucida* Vill.**	Orophitic-European	Mt. Cimone	2110
*Crassulaceae*	***Sempervivum montanum* L. subsp. *montanum***	Orophitic-European	Mt. Cimone	1970
*Caryophyllaceae*	***Silene acaulis* subsp. *bryoides* (Jord.) Nyman**	Circumboreal	Mt. Cimone	2030
*Caryophyllaceae*	***Silene suecica* (Lodd.) Greuter & Burdet**	Circumboreal	Mt. Prado	2035
*Asteraceae*	***Solidago virgaurea* subsp. *minuta* (L.) Arcang.**	Eurosiberian	Mt. Cimone	1875
*Fabaceae*	***Trifolium alpinum* L.**	Orophitic-European	Mt. Cimone	1895
*Ericaceae*	***Vaccinium uliginosum* L. subsp. *microphyllum* (Lange) Tolm.** (= *Vaccinium gaultherioides* Bigelow)	Circumboreal	Mt. Cimone	1875
*Ericaceae*	***Vaccinium myrtillus* L.**	Circumboreal	Mt. Cimone	1875

Information on species chorotype was adapted from Alessandrini *et al*. 2003 [42]. For each species we reported the site and the elevation of the collected population.

### Germination phenology under simulated weekly temperatures

Laboratory treatments involved sowing three replicates of 30 seeds per species for each of the three temperature treatments described below on 1% distilled water–agar held in 90 mm diameter Petri dishes. Treatments were carried out in temperature and light-controlled incubators (LMS Ltd, Sevenoaks, UK) using a 12-h daily photoperiod. Light was provided by cool white fluorescent tubes, with a photosynthetically active radiation of 40–55 μmol m^-2^ s^-1^(400–700 nm).

**Fig 1:**
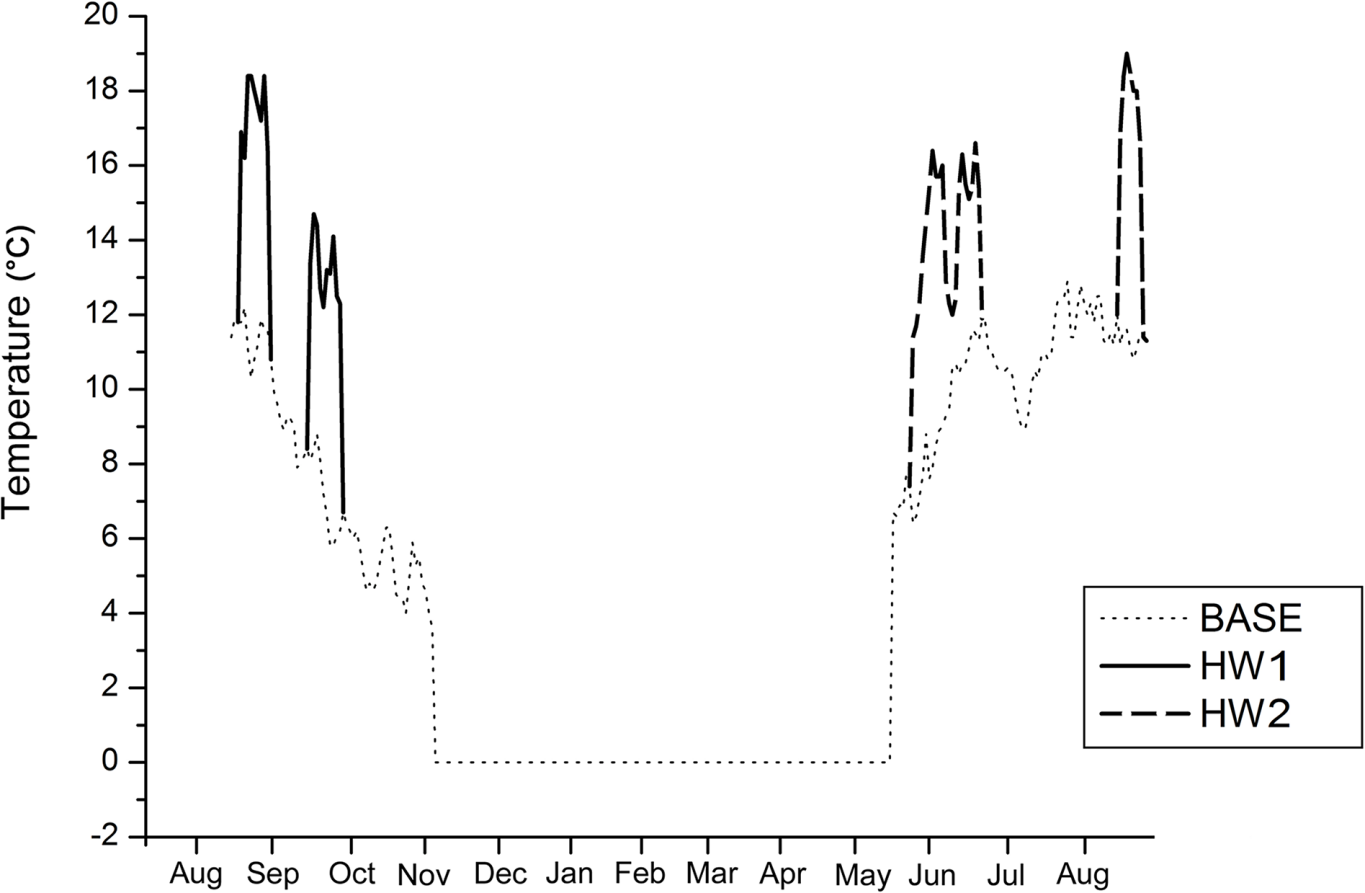
Mean daily air temperatures (°C) at the site where most of the species were collected (Monte Cimone; dot line) in the period 1999–2011, and the two heat wave episodes that occurred in autumn 2011 (HW1; continuous line) and spring 2003 (HW2; dashed line).

From the time of collection (mid-August), seeds were exposed to three temperature treatments: BASE, i.e. mean weekly changes that were recorded at the species growing site between 1999 and 2011; HW1, i.e. a simulation of heat waves that occurred between 20^th^ and 30^th^ August 2011 and between 8^th^ and 18^th^ September 2011 (hereafter referred to as autumn heat waves); and HW2, i.e. a simulation of heat waves that occurred between 7^th^ and 26^th^ June 2003 and between 8^th^ and 14^th^ August 2003 (hereafter referred to as spring heat waves) (Table 2; Fig 1). Each treatment derived from air temperature measurements that were taken at 2m above ground, at hourly intervals, between 1999 and 2011, by the Italian Climate Observatory “Ottavio Vittori” (ICO-OV), managed by ISAC-CNR (Bologna) (Fig 1) and located near the species growing site on the top of Mt. Cimone. ICO-OV is part of the Global Atmospheric Watch (GAW) programme by the World Meteorological Organization (WMO) and SHARE (Station at High Altitude for Research on the Environment) project. Data from this station were chosen because they represented the most reliable temperature measurements available (i.e. a complete 12-year series) close to the species growing site. As an incubation temperature, we used constant mean day (8am-8pm) and night (8pm-8am) temperatures, because of the subtle temperature differences (i.e. <5°C) between day and night recorded during heat waves. Furthermore, in order to simulate the insulating effects of snow cover, winter temperatures were kept at a constant 0°C for 7 months (according to the climatic data from ICO-OV), in complete darkness (from mid-October to the end of May). Darkness prevents germination, at least in positive photoblastic species (e.g. *Luzula alpinopilosa*), although the light requirement of most of the species is unknown. Seeds were regularly checked for germination at 5-day intervals.

**Table 2:** Temperatures during the different days of the incubation treatments and the equivalent week (w.) of the year. The two HW treatments are highlighted in bold.

		Temperature treatments (°C)		
Day of the experiments	Equivalent time of the year	BASE	HW 1	HW 2
**1–6**	3^rd^ w. August	11°C	11°C	11°C
**7–17**	4^th^ w. August	11°C	**17**°**C**	11°C
**18–25**	1^st^ w. September	7°C	7°C	7°C
**26–36**	2^nd^-3^rd^ w. September	7°C	**13**°**C**	7°C
**37–42**	4^th^ w. September	7°C	7°C	7°C
**43–53**	1^st^ w. October	5°C	5°C	5°C
**54–258**	2^nd^ w. October- 4^th^ w. May	0°C	0°C	0°C
**259–305**	1^st^ w. June	9°C	9°C	9°C
**306–326**	2^nd^-4^th^ w. June	9°C	9°C	**15°C**
**327–360**	1^st^ w. July-1^st^ w. August	11°C	11°C	11°C
**361–367**	2^nd^ w. August	11°C	11°C	**19°C**

### Water potential

Laboratory germination tests at different water potentials were set up to simulate decreasing soil water availabilities that could be related to HW1. HW2 was not included in these experiments, since it occurred a few days after snowmelt, when soil moisture was high. Seeds belonging to 10 previously tested species, which showed an increase in germination during autumn heat waves (HW1) under full hydration (i.e. 0 MPa), were subjected to water potentials of approx. -0.1, -0.2, -0.4 and -0.8 MPa and then exposed to BASE and HW1 temperature treatments. In each case, seeds were sown on filter papers soaked in a solution of polyethylene glycol 6000 (PEG) (Alfa Aesar GmbH & Co KG, Germany), at a concentration appropriate to the intended treatment [44]. A small portion of solution (3 ml) was added to the filter papers every time germination was monitored, to avoid changes in concentration due to solution evaporation. For each test, the number of seeds and replications, light conditions and observations were as described above.

### Data analysis

Species with a germination percentage lower than 5% in all temperature treatments were excluded from statistical analysis (n = 5). All analyses were carried out using the R software (version 3.1.1) [45]. We estimated success of seed germination during autumn, summer and at the end of each treatment using three generalized linear mixed effect models (GLMMs) for binary data with binomial error distribution. Treatments (BASE, HW1 and HW2) were used as fixed effects, while replicates and species identity were included as random effects in each model. We performed a post-hoc multiple comparison between fixed effects using the “glht” function in the “multcomp” package in R [46], computing the Tukey's honest significance test. We also used generalized linear models (GLM) for binary data with binomial error distribution to estimate the variation between treatments occurring for each tested species (n = 48), computing the Tukey's honest significance test.

The same GLMM, GLM and multiple comparison described above were applied to estimate seed germination at different treatments and at different water potentials, both at global and species level, respectively. We used GLM to estimate the success of germination at the three different treatments for each chorotype class. We finally performed a chi-square test for each species separately to compare the number of seeds that germinated in autumn between BASE and HW1 conditions.

## Results

### Effects of autumn heat waves on germination phenology

**Fig 2:**
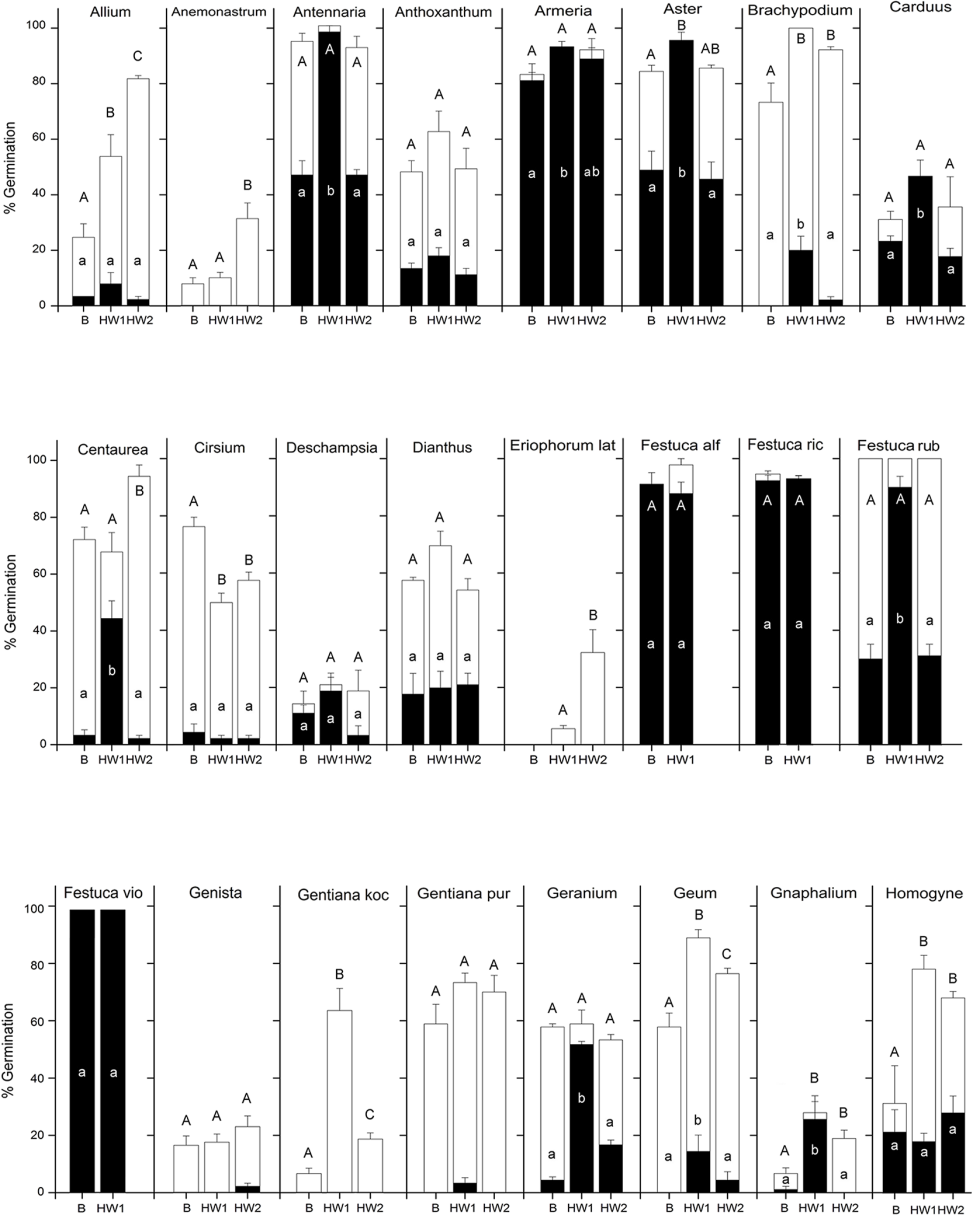
Cumulative germination percentage (means ± s.e.) of each species (*Allium*-*Homogyne*) under three temperature treatments at the end of autumn (black columns) and at the end of summer (white columns). Winter germination is not shown since no seeds germinate during cold stratification period. Final germination is given by the sum of black and white. Lowercase letters indicate significant differences of germination at P<0.05 level (Tukey's honest significance test) in autumn. Capital letters indicate significant differences of final germination at P<0.05 level (Tukey's honest significance test) (i.e. sum of autumn and spring/summer germination).

**Fig 3:**
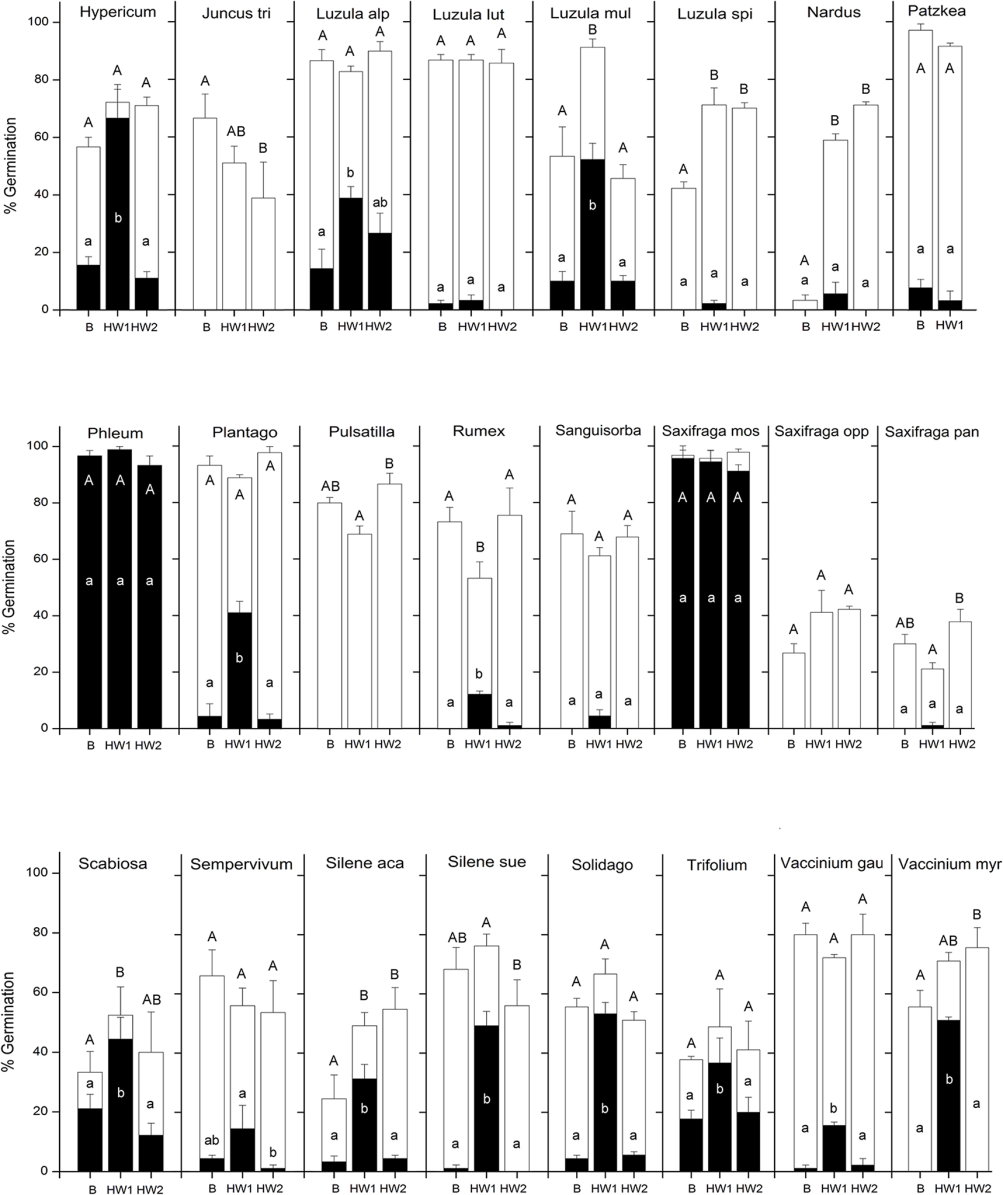
Cumulative germination percentage (means ± s.e.) of each species (*Hypericum*-*Vaccinium myr*) under three temperature treatments at the end of autumn (black columns) and at the end of summer (white columns). Winter germination is not shown since no seeds germinate during cold stratification period. Final germination is given by the sum of black and white. Lowercase letters indicate significant differences of germination at P<0.05 level (Tukey's honest significance test) in autumn. Capital letters indicate significant differences of final germination at P<0.05 level (Tukey's honest significance test) (i.e. sum of autumn and spring/summer germination).

Germination response just after seed dispersal in late-summer/autumn (16^th^ August-10^th^ October, Table 2) varied across species and temperature treatments (Figs 2 and 3 and S1 Table) and occurred within the first month after sowing. In particular, in the absence of heat waves (BASE, Table 2), seed germination was low (0–25%) across most of the species, with only eight species showing more than 30% germination (see Figs 2 and 3 and S1 Table). However, the increase in autumn temperatures according to the HW1 scenario (Table 2) elicited a significant increase in germination in 23 of the 48 species included in the analysis (Figs 2 and 3 and S1 Table). In some species, germination more than doubled: *Antennaria* (from 47% to 98%), *Aster* (from 47% to 96%), *Carduus* (from 23% to 47%), *Centaurea* (from 3% to 44%), *Festuca rubra* (from 30% to 90%), *Geranium* (from 10% to 52%), *Gnaphalium* (from 1% to 26%), *Hypericum* (from 13% to 67%), *Luzula multiflora* (from 10% to 52%), *Plantago* (from 3% to 41%), *Scabiosa* (from 17% to 44%); *Silene acaulis* (from 3% to 31%), *Silene suecica* (from 1 to 49%), *Solidago* (from 5% to 53%) and *Vaccinium myrtillus* (from 0 to 51%). However, only a moderate increase in the germination percentage was observed in other species (see S1 Table). When seeds were transferred to the winter temperature (0°C), germination stopped in each of the tested treatments. Germination resumed when seeds were transferred to spring conditions, showing significant differences between BASE and HW1 (Table 3). In particular, summer germination increased significantly for seeds experiencing HW1 in eight species, but it reduced in another five species (S1 Table). Considering the germination at the end of each treatment (i.e. the sum of summer/autumn and spring/summer emergence), there were significant differences between BASE and HW1 (Table 3), with the latter resulting in a significant increase of germination in 12 species and a significant decrease in two species (Figs 2 and 3 and S1 Table).

**Table 3:** Results of the generalized linear mixed effects models (GLMMs) on the effects of treatments (HW1, HW2 and B) on autumn, summer and final seed germination.

Treatment	Germination AUTUMN				Germination SUMMER				Germination FINAL			
	Estimate	Std.Err	Z-value	P-value	Estimate	Std.Err	Z-value	P-value	Estimate	Std.Err	Z-value	P-value
HW1×B	1.699	0.077	21.855	**<0.001**	0.169	0.058	2.879	**0.011**	0.538	0.053	10.141	**<0.001**
HW2×B	-0.069	0.086	-0.805	0.699	0.548	0.056	9.767	**<0.001**	0.474	0.053	8.909	**<0.001**
HW1×HW2	1.769	0.080	22.108	**<0.001**	-0.379	0.059	-6.372	**<0.001**	0.063	0.053	1.185	0.462

### Effects of spring heat waves on germination phenology

In four species, the effects of spring heat waves (Table 2) was not tested because more than 90% of seeds germinated in autumn at the BASE temperature treatment (*Festuca alfrediana*, *F*. *riccerii* and *F*. *violacea*), or because only a few seeds were available (*Patzkea paniculata*). In general, significant differences in summer germination were observed between BASE and HW2 temperature treatments (Table 3). Germination occurred within the first month after winter stratification and increased significantly in 15 species experiencing HW2, compared to those under BASE treatment, but it reduced in two species (S1 Table). Considering the final germination (i.e. sum of summer/autumn and spring/summer emergence), there were significant differences between BASE and HW2 (Table 3), with germination increasing significantly in HW2 compared to BASE in 10 species (Figs 2 and 3 and S1 Table).

Comparing summer germination in HW1 and HW2, significant differences were also reported (Table 3). Summer germination increased significantly in HW2 compared to HW1 in 14 species, but the opposite was observed in four species (Figs 2 and 3 and S1 Table).

There were no significant differences in final germination between HW1 and HW2 (Table 3). However, considering the single-species models, final germination was higher in HW2 in seven species compared to HW1, while the opposite was observed in four species (Figs 2 and 3 and S1 Table).

### Germination at different water potentials in autumn

**Fig 4:**
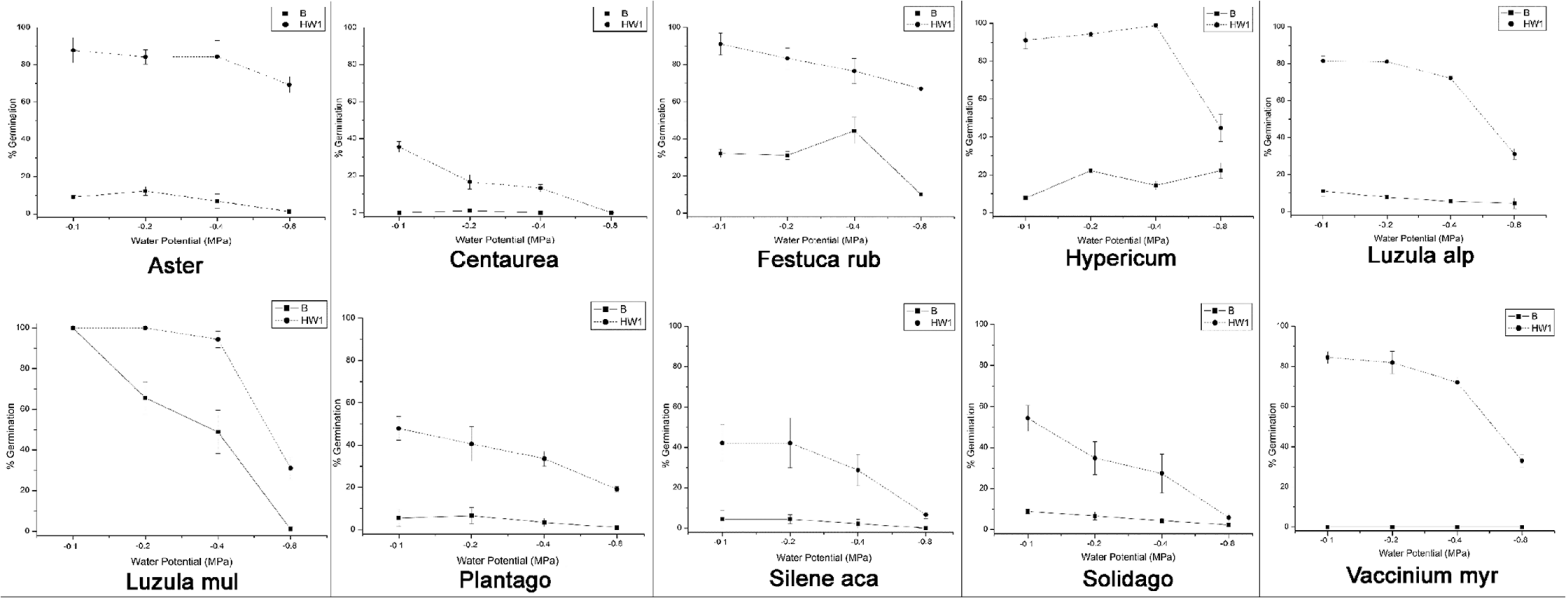
Autumn germination percentage (means ± s.e.) of ten species at different water potentials (MPa) under BASE treatment (B) and autumn heat waves (HW1).

Seeds of the 10 species that showed a significant germination response to autumn heat waves (HW1) were further exposed to different water potentials. Despite the large inter-species variation, seed germination decreased with decreasing water potential, both in HW1 and in BASE temperature treatments (Table 4 and Fig 4). However, in three species (*Aster*, *Festuca rubra* and *Hypericum*) more than 50% of seeds were able to germinate under HW1 even at -0.8 MPa. Furthermore, in six species (*Aster*, *Festuca rubra*, *Hypericum*, *Luzula alpinopilosa*, *Luzula multiflora* and *Vaccinium myrtillus*) seed germination under HW1 at -0.4 MPa was higher than 70% (Table 5 and Fig 4).

**Table 4:** Results of the generalized linear mixed effects models (GLMMs) on the effect of water potentials (WP) and treatment on seed germination.

	Estimate	Std. Error	Z-value	Df	P-value
Treatment	2.97081	0.13803	21.524	1	**<0.001**
WP	-0.23185	0.03416	-6.786	3	**<0.001**
Treatment×WP	-0.09485	0.04238	-2.238	3	**0.0252**

**Table 5:** Results of the chi-square test for differences in germination at different water potentials within treatments. Within each treatment the effect of four water potentials (-0.1 MPa, -0.2 MPa, -0.4 MPa, -0.8MPa) was tested.

Species	Treatments	Germination AUTUMN		
		Df	Chisq	P-value
***Aster alpinus***	Base	4	67.310	**<0.001**
	HW1	4	19.943	**<0.001**
***Centaurea nervosa***	Base	4	0.9278	0.920
	HW1	4	28.131	**<0.001**
***Festuca nigrescens***	Base	4	23.279	**<0.001**
	HW1	4	21.857	**<0.001**
***Hypericum richeri* subsp. *richeri***	Base	4	9.053	0.059
	HW1	4	83.512	**<0.001**
***Luzula alpinopilosa* subsp. *alpinopilosa***	Base	4	7.204	0.125
	HW1	4	76.594	**<0.001**
***Luzula multiflora* subsp. *multiflora***	Base	4	65.503	**<0.001**
	HW1	4	69.774	**<0.001**
***Plantago alpina* subsp. *alpina***	Base	4	3.461	0.483
	HW1	4	16.872	**0.004**
***Silene acaulis* subsp. *bryoides***	Base	4	0.442	0.978
	HW1	4	33.806	**<0.001**
***Solidago virgaurea* subsp. *minuta***	Base	4	4.211	0.378
	HW1	4	51.818	**<0.001**
***Vaccinium myrtillus***	Base	4	0.000	1
	HW1	4	58.073	**<0.001**

### Germination response in different chorotypes

Autumn germination under HW1 increased significantly compared to BASE in Arctic-alpine, Circumboreal, Eurosiberian and Orophitic-European species (Table 6). In Endemic species, the differences were just below the threshold of significance, whilst in Eurasiatic and Subcosmopolitan species there were no significant differences between HW1 and BASE (Table 6). Summer germination increased significantly in HW1 compared to BASE in Orophitic-European species, while it decreased significantly in Circumboreal (Table 6). Final germination increased significantly in HW1 compared to BASE in Arctic-alpine, Circumboreal, Eurosiberian and Orophitic-European species (Table 6). Summer germination increased significantly once more in Euroasiatic, Circumboreal, Eurosiberian, Orophitic-European and Subcosmopolitan species in HW2 compared to BASE, while in Arctic-alpine and Endemic species there were no differences in summer germination between temperature treatments (Table 6). Final germination between BASE and HW2 was significantly different, with a general increase in the latter, for Circumboreal, Eurosiberian, Eurasiatic and Orophitic-European species (Table 6).

**Table 6:** Results of the generalized linear mixed effects models (GLMMs) on the effects on biogeographic distribution (chorology) on seed germination between control and heat waves treatments.

Chorotype	Treatments	Germination AUTUMN				Germination SUMMER			Germination FINAL				
		Estimate	Std.Err	Z-value	P-value	Estimate	Std.Err	Z-value	P-value	Estimate	Std.Err	Z-value	P-value
**Arctic-Alpine**	B×HW1	1.063	0.220	4.825	**<0.001**	0.043	0.288	0.152	0.879	0.650	0.241	2.695	**0.007**
	B×HW2	-	-	-	-	0.138	0.251	0.550	0.582	0.050	0.223	0.224	0.823
**Circumboreal**	B×HW1	1.701	0.118	14.345	**<0.001**	-0.257	0.087	-2.956	**0.003**	0.309	0.077	3.974	**<0.001**
	B×HW2	-	-	-	-	0.326	0.080	4.046	**<0.001**	0.322	0.077	4.130	**<0.001**
**Endemic**	B×HW1	0.300	0.149	2.011	**0.044**	-0.027	0.221	-0.123	0.902	0.139	0.199	0.697	0.486
	B×HW2	-	-	-	-	0.200	0.222	0.898	0.369	-0.098	0.206	-0.475	0.635
**Eurasiatic**	B×HW1	0.130	0.361	0.360	0.719	0.426	0.252	1.689	0.091	0.374	0.224	1.670	0.094
	B×HW2	-	-	-	-	0.685	0.248	2.762	**0.005**	0.605	0.222	2.726	**0.006**
**Euro-siberia**	B×HW1	2.546	0.434	5.865	**<0.001**	0.047	0.157	0.303	0.762	0.364	0.157	4.955	**0.016**
	B×HW2	-	-	-	-	0.775	0.157	4.919	**<0.001**	0.778	0.151	2.409	**<0.001**
**Orophitic-European**	B×HW1	0.646	0.077	8.301	**<0.001**	0.334	0.082	4.047	**<0.001**	0.543	0.074	7.324	**<0.001**
	B×HW2	-	-	-	-	0.326	0.079	4.091	**<0.001**	0.197	0.073	2.682	**0.007**
**Subcosmopolitan**	B×HW1	0.622	0.430	1.446	0.148	-0.324	0.927	-0.350	0.726	0.460	0.395	1.164	0.244
	B×HW2	-	-	-	-	1.593	0.656	2.426	**0.015**	0.321	0.403	0.798	0.425

## Discussion

Although there have been previous studies on the effects of climate warming on seed germination in alpine species, they focused on continuous seasonal temperature warming [33,36,37,38] instead of short term heat waves. The results reported here show that in the absence of heat waves (i.e. the BASE treatment), seed germination mainly occurred in spring, after seeds had experienced late-summer, autumn and winter seasons. Indeed, seed germination in alpine plants tends to occur shortly after snowmelt in spring [47,48]. Conversely, in autumn, germination significantly increased after heat waves in half the species (23 out of 48), indicating that heat waves may affect the timing of germination. Supporting this view, Mondoni *et al*. [34] cautioned that warming may lead to a shift from mostly spring emergence to autumn emergence in several glacier foreland species, particularly among those with non-dormant or conditionally dormant seeds (*sensu* Baskin & Baskin, [32]). However, we observed that in some cases autumn heat waves had no impact or only a minor impact on germination in autumn (probably due to a deep dormancy state), but it significantly increased the subsequent summer germination, as shown in *Allium*, *Brachypodium*, *Gentiana kochiana*, *Geum*, *Homogyne*, *Luzula multiflora*, *Luzula spicata* and *Nardus*. On the other hand, autumn heat waves reduced summer germination in *Centaurea*, *Cirsium*, *Hypericum*, *Rumex* and *Solidago*. It is therefore not possible to generalize across species about the effects that autumn heat waves have on germination. Furthermore, even when autumn emergence is not affected (i.e. probably due to a deep dormancy state), potential changes in summer emergence cannot be ruled out.

We observed that when heat waves occurred just after snow melt, summer germination increased in 15 out of 48 species, confirming that high temperature *per se* may have positive effects on seed germination [33]. This finding is supported by the germination results observed at the end of the treatments (the sum of summer/autumn and spring/summer germination): there was a significant increase in seed germination in HW1 and HW2 compared to BASE, but there were no significant differences between HW1 and HW2. Although heat waves have been shown to have positive effects on seed germination *per se*, implications on subsequent seedling survival may depend on when these events occur. For example, spring heat waves may enhance seedling recruitment as the timing of emergence is similar to that experienced in the absence of heat waves (i.e. in spring after snow melt), but the proportion of the emerged seedlings is higher. However, the advanced snow melting and the depletion of soil moisture that are often associated to heat waves may result in a rapid die off of the seedlings [49]. Conversely, autumn heat waves could alter germination phenology by eliciting an anomalous germination immediately after seed dispersal, whose effects on seedling survival remains unknown.

Autumn emergence could have major implications for species that are currently adapted to emerge in spring [34]. In this regard, the phenology of germination of some *Festuca* species (Poaceae) suggests that seedling survival during winter is possible. Supporting this view, Marcante *et al*. [50] demonstrated that in some pioneer species, seedlings may exhibit a frost resistance (-5.6°C) in the field. Moreover, chances of seedling survival are also linked to air temperature and snow cover. Air temperatures during winter can drop below -15°C, whilst the soil is usually covered by snow, which moderates temperatures at about 0°C [47]. However, future changes in snowfall and precipitation patterns that could reduce the duration and extent of snow cover [51] would alter the thermal protection of the snow-pack on seedlings [52], thus making them more susceptible to late spring or early summer frost events [53]. Although soil moisture depletion is generally rare in alpine environments [47], one of the possible consequences of heat waves is the decrease of water content of the near-surface soil [54], where seeds usually germinate. In our study, the seed germination of several species (*Hypericum*, *Luzula alpinopilosa*, *Luzula multiflora*, *Plantago*, *Silene acaulis*, *Solidago* and *Vaccinium myrtillus*) strongly reduced at low water potential (-0.8 MPa, Fig 4), perhaps as a response to prevent germination in drying soils [55]. However, most species were able to germinate under conditions that represented moderate drought (-0.4 MPa; Fig 4); furthermore, in two species (*Aster* and *Festuca rubra*) seed germination was higher than 70% even at the driest condition tested (-0.8 MPa). Hence, when heat waves are associated with a decrease in soil moisture levels, we cannot rule out the possibility that some species may be able to germinate. Moreover, storms and rainfall can be frequent events, although they are stochastic in mountain areas, as reported for our study area in summer and autumn [56], providing a water supply for germination. Nevertheless, prolonged water stress may compromise seedling viability, since drought conditions usually exacerbate the negative effect of heat stress on plant growth [57]. Plant mortality in juvenile stages may result in negative impacts on the genetic variability of the populations, and this could have possible consequences on natural selection.

At plant family level, some of the *Poaceae* and *Juncaceae* showed similar germination regardless of treatment. Grasses are generally predicted to be favoured by climate change in arctic and alpine habitats [37,58], which is probably due to their increased heat tolerance in both adults [59] and seedlings [60]. Moreover, their higher tissue turnover and photosynthetic system enable them to respond more rapidly to environmental manipulations than other plant functional types [61,62]. If we consider seed germination, this hypothesis is partially confirmed by previous experiments [34] and by our results, which show that 4 out of 10 *Poaceae* tested adapted to autumn germination and more than half (12 out of 16) of the graminoids (*Poaceae*, *Juncaceae* and *Cyperaceae*) showed no germination differences between treatments (Figs 2 and 3 and S1 Table).

Interestingly, in our study the seed germination of cold-adapted chorotypes (Arctic-alpine, Circumboreal, Eurosiberian and Orophitic, Endemic to the Northern Apennines) was significantly higher during autumn heat waves than the germination of widely distributed species (Subcosmopolitan and Eurasiatic). This difference suggests that varying environmental conditions experienced by the species in the past may have affected their germination responses. Assuming that selection pressure should favour individuals that decrease the probability of encountering unfavourable growth conditions following germination [63], we interpret such results as the consequence of the selective pressure exerted by cold winter temperatures on seedlings that emerged during mild autumns in widely-distributed species. In contrast, in cold environments where autumn is consistently cool, species may have not developed mechanisms to prevent seed germination during heat waves. Therefore, in a climatic scenario with increased frequency and intensity of heat waves, we would expect a strong selective pressure on cold adapted chorotypes.

In conclusion, our results suggest that short term periods of high temperatures, like heat waves, can enhance seed germination even immediately after seed dispersal and even under moderate drought. These results are based on lab simulations of air temperatures and thus there is still uncertainty about the temperatures that seeds may actually experience in the field. The response of seed germination to heat waves is species-specific. Consistent responses across chorotypes suggest that heat waves may have important consequences on seed germination and, in turn, selectively affect seedling recruitment. These differences may result in range shift and/or changes in local species dominance in montane and alpine plant communities and may have considerable effects on their genetic characteristics and adaptation potential.

## Supporting Information

S1 Table Results of generalized linear mixed effects models (GLMMs) on the effects of heat waves on seed germination of the tested species. (DOCX)
