# Supplementary material for: Effects of Autumn and Spring Heat Waves on Seed Germination of High Mountain Plants
Source: PLoS One. 2015 Jul 21;10(7):e0133626. doi: 10.1371/journal.pone.0133626 (PMC4509759; doi:10.1371/journal.pone.0133626)
Supplement: S1 Table — (DOCX) [file pone.0133626.s001.docx]

**Table S1.** Results of generalized linear mixed effects models (GLMMs) on the effects of heat waves on seed germination of the tested species.

| **Species** | **Treatments** | **Germination AUTUMN** | | | | **Germination SUMMER** | | | | | **Germination FINAL** | | | | | |
| --- | --- | --- | --- | --- | --- | --- | --- | --- | --- | --- | --- | --- | --- | --- | --- | --- |
|  |  | Estimate | Std.Err | Z-value | P-value | | Estimate | Std.Err | Z-value | P-value | | Estimate | Std.Err | Z-value | P-value |  |
| ***Allium schoenoprasum*** | HW1×B | 0.894 | 0.706 | 1.265 | 0.411 | | 1.250 | 0.339 | 3.680 | **<0.001** | | 1.261 | 0.323 | 3.898 | **<0.001** |  |
|  | HW2×B | -0.416 | 0.925 | -0.450 | 0.892 | | 2.704 | 0.374 | 7.221 | **<0.001** | | 2.585 | 0.364 | 7.098 | **<0.001** |  |
|  | HW1×HW2 | 1.311 | 0.816 | 1.606 | 0.239 | | -1.453 | 0.348 | -4.176 | **<0.001** | | -1.323 | 0.342 | -3.867 | **<0.001** |  |
| ***Anemonastrum narcissiflorum*** | HW1×B | - | - | - | - | | 0.275 | 0.527 | 0.522 | 0.858 | | 0.275 | 0.527 | 0.522 | 0.858 |  |
|  | HW2×B | - | - | - | - | | 1.678 | 0.454 | 3.690 | **<0.001** | | 1.678 | 0.454 | 3.690 | **<0.001** |  |
|  | HW1×HW2 | - | - | - | - | | -1.402 | 0.418 | -3.349 | **0.002** | | -1.402 | 0.418 | -3.349 | **0.002** |  |
| ***Antennaria dioica*** | HW1×B | 3.917 | 0.745 | 5.254 | **<0.001** | | -1.458 | 1.312 | -1.111 | 0.492 | | 1.655 | 1.105 | 1.496 | 0.282 |  |
|  | HW2×B | 0.000 | 0.298 | 0.000 | 1 | | -0.384 | 0.624 | -0.614 | 0.787 | | -0.360 | 0.605 | -0.594 | 0.799 |  |
|  | HW1×HW2 | 3.917 | 0.745 | 5.254 | **<0.001** | | -1.074 | 1.291 | -0.832 | 0.671 | | 2.015 | 1.079 | 1.866 | 0.141 |  |
| ***Anthoxanthum alpinum*** | HW1×B | 0.340 | 0.414 | 0.820 | 0.690 | | 0.578 | 0.328 | 1.761 | 0.182 | | 0.587 | 0.302 | 1.940 | 0.127 |  |
|  | HW2×B | -0.207 | 0.456 | -0.454 | 0.892 | | 0.113 | 0.323 | 0.351 | 0.933 | | 0.044 | 0.298 | 0.149 | 0.987 |  |
|  | HW1×HW2 | 0.547 | 0.434 | 1.262 | 0.416 | | 0.464 | 0.324 | 1.430 | 0.325 | | 0.543 | 0.302 | 1.794 | 0.171 |  |
| ***Armeria marginata*** | HW1×B | 1.181 | 0.501 | 2.358 | **0.047** | | 0.223 | 1.316 | 0.169 | 0.984 | | 1.029 | 0.508 | 2.024 | 0.105 |  |
|  | HW2×B | 0.622 | 0.430 | 1.446 | 0.314 | | 1.167 | 1.021 | 1.143 | 0.441 | | 0.863 | 0.484 | 1.781 | 0.174 |  |
|  | HW1×HW2 | 0.559 | 0.539 | 1.037 | 0.551 | | -0.944 | 1.281 | -0.736 | 0.739 | | 0.166 | 0.577 | 0.287 | 0.955 |  |
| ***Aster alpinus*** | HW1×B | 3.112 | 0.553 | 5.626 | **<0.001** | | -2.212 | 1.163 | -1.902 | 0.126 | | 1.376 | 0.588 | 2.339 | **0.049** |  |
|  | HW2×B | -0.133 | 0.298 | -0.447 | 0.891 | | 0.191 | 0.455 | 0.421 | 0.893 | | 0.087 | 0.417 | 0.208 | 0.975 |  |
|  | HW1×HW2 | 3.246 | 0.553 | 5.865 | **<0.001** | | -2.404 | 1.163 | -2.066 | 0.087 | | 1.289 | 0.592 | 2.174 | 0.073 |  |
| ***Brachypodium genuense*** | HW1×B | 3.102 | 1.039 | 2.984 | **0.007** | | 3.265 | 1.034 | 3.155 | **0.003** | | 3.477 | 1.033 | 3.364 | **0.001** |  |
|  | HW2×B | 0.704 | 1.233 | 0.570 | 0.829 | | 1.436 | 0.460 | 3.120 | **0.003** | | 1.461 | 0.460 | 3.175 | **0.002** |  |
|  | HW1×HW2 | 2.397 | 0.762 | 3.146 | **0.003** | | 1.828 | 1.081 | 1.690 | 0.194 | | 2.015 | 1.079 | 1.866 | 0.137 |  |
| ***Carduus defloratus* subsp. *carlinifolius*** | HW1×B | 1.056 | 0.326 | 3.232 | **0.003** | | -1.668 | 1.086 | -1.536 | 0.259 | | 0.661 | 0.310 | 2.129 | 0.084 |  |
|  | HW2×B | -0.341 | 0.371 | -0.919 | 0.626 | | 0.893 | 0.488 | 1.828 | 0.130 | | 0.200 | 0.316 | 0.632 | 0.802 |  |
|  | HW1×HW2 | 1.397 | 0.347 | 4.024 | **<0.001** | | -2.562 | 1.049 | -2.442 | **0.035** | | 0.461 | 0.305 | 1.511 | 0.285 |  |
| ***Centaurea nervosa*** | HW1×B | 3.144 | 0.624 | 5.035 | **<0.001** | | -1.231 | 0.371 | -3.311 | **0.002** | | -0.211 | 0.325 | -0.650 | 0.787 |  |
|  | HW2×B | -0.416 | 0.925 | -0.450 | 0.890 | | 1.901 | 0.517 | 3.671 | **<0.001** | | 1.877 | 0.516 | 3.632 | **<0.001** |  |
|  | HW1×HW2 | 3.561 | 0.745 | 4.774 | **<0.001** | | -3.132 | 0.542 | -5.775 | **<0.001** | | -2.089 | 0.512 | -4.077 | **<0.001** |  |
| ***Cirsium bertolonii*** | HW1×B | -0.716 | 0.879 | -0.814 | 0.692 | | -1.175 | 0.329 | -3.568 | **0.001** | | -1.189 | 0.326 | -3.644 | **<0.001** |  |
|  | HW2×B | -0.716 | 0.879 | -0.814 | 0.692 | | -0.855 | 0.330 | -2.587 | **0.026** | | -0.875 | 0.328 | -2.669 | **0.020** |  |
|  | HW1×HW2 | 0.000 | 1.011 | 0.000 | 1 | | -0.319 | 0.302 | -1.055 | 0.541 | | -0.313 | 0.299 | -1.045 | 0.547 |  |
| ***Deschampsia cespitosa* subsp. *cespitosa*** | HW1×B | 0.622 | 0.430 | 1.446 | 0.310 | | -0.324 | 0.927 | -0.349 | 0.933 | | 0.460 | 0.395 | 1.163 | 0.474 |  |
|  | HW2×B | -1.287 | 0.676 | -1.904 | 0.132 | | 1.593 | 0.656 | 2.426 | **0.038** | | 0.321 | 0.403 | 0.797 | 0.704 |  |
|  | HW1×HW2 | 1.910 | 0.646 | 2.956 | **0.008** | | -1.918 | 0.774 | -2.477 | **0.034** | | 0.139 | 0.373 | 0.372 | 0.926 |  |
| ***Dianthus deltoides*** | HW1×B | 0.145 | 0.381 | 0.380 | 0.923 | | 0.564 | 0.336 | 1.677 | 0.213 | | 0.533 | 0.313 | 1.700 | 0.204 |  |
|  | HW2×B | 0.213 | 0.377 | 0.564 | 0.838 | | -0.258 | 0.334 | -0.772 | 0.719 | | -0.135 | 0.300 | -0.450 | 0.894 |  |
|  | HW1×HW2 | -0.068 | 0.368 | -0.184 | 0.981 | | 0.823 | 0.342 | 2.406 | **0.042** | | 0.669 | 0.312 | 2.140 | 0.081 |  |
|  |  |  |  |  |  | |  |  |  |  | |  |  |  |  |  |
| **Species** | **Treatments** | **Germination AUTUMN** | | | | | **Germination SUMMER** | | | | | **Germination FINAL** | | | |  |
|  |  | Estimate | Std.Err | Z-value | P-value | | Estimate | Std.Err | Z-value | P-value | | Estimate | Std.Err | Z-value | P-value |  |
| ***Eriophorum latifolium*** | HW1×B | - | - | - | - | | 1.655 | 1.105 | 1.496 | 0.277 | | 1.655 | 1.105 | 1.496 | 0.277 |  |
|  | HW2×B | - | - | - | - | | 3.745 | 1.030 | 3.633 | **<0.001** | | 3.745 | 1.030 | 3.633 | **<0.001** |  |
|  | HW1×HW2 | - | - | - | - | | -2.089 | 0.512 | -4.077 | **<0.001** | | -2.089 | 0.512 | -4.077 | **<0.001** |  |
| ***Festuca alfrediana*** | HW1×B | -0.355 | 0.490 | -0.724 | 0.468 | | 0.980 | 0.978 | 1.001 | 0.316 | | 0.299 | 0.778 | 0.384 | 0.700 |  |
| ***Festuca rubra* subsp. *commutata*** | HW1×B | 3.044 | 0.419 | 7.249 | **<0.001** | | -2.047 | 1.463 | -1.399 | 0.341 | | 0.000 | 1.422 | 0.000 | 1 |  |
|  | HW2×B | 0.052 | 0.323 | 0.161 | 0.985 | | -0.016 | 1.425 | -0.011 | 0.999 | | 0.000 | 1.422 | 0.000 | 1 |  |
|  | HW1×HW2 | 2.992 | 0.418 | 7.146 | **<0.001** | | -2.031 | 1.463 | -1.388 | 0.347 | | 0.000 | 1.422 | 0.000 | 1 |  |
| ***Festuca paniculata*** | HW1×B | -0.894 | 0.706 | -1.265 | 0.205 | | -1.411 | 0.806 | -1.750 | 0.080 | | -1.456 | 0.805 | -1.809 | 0.070 |  |
| ***Festuca riccerii*** | HW1×B | 0.194 | 0.624 | 0.310 | 0.755 | | -0.916 | 1.396 | -0.656 | 0.511 | | -0.234 | 0.687 | -0.341 | 0.732 |  |
| ***Festuca violacea* subsp. *puccinelli*** | HW1×B | - | - | - | - | | - | - | - | - | | - | - | - | - |  |
| ***Genista radiata*** | HW1×B | - | - | - | - | | 0.077 | 0.394 | 0.197 | 0.978 | | 0.077 | 0.394 | 0.197 | 0.978 |  |
|  | HW2×B | 0.704 | 1.233 | 0.570 | 0.834 | | 0.319 | 0.383 | 0.833 | 0.681 | | 0.419 | 0.376 | 1.113 | 0.505 |  |
|  | HW1×HW2 | -0.704 | 1.233 | -0.570 | 0.834 | | -0.241 | 0.378 | -0.639 | 0.798 | | -0.341 | 0.371 | -0.919 | 0.627 |  |
| ***Gentiana kochiana*** | HW1×B | - | - | - | - | | 3.233 | 0.476 | 6.786 | **<0.001** | | 3.233 | 0.476 | 6.786 | **<0.001** |  |
|  | HW2×B | - | - | - | - | | 1.181 | 0.501 | 2.358 | **0.046** | | 1.181 | 0.501 | 2.358 | **0.046** |  |
|  | HW1×HW2 | - | - | - | - | | 2.051 | 0.347 | 5.898 | **<0.001** | | 2.051 | 0.347 | 5.898 | **<0.001** |  |
| ***Gentiana purpurea*** | HW1×B | 1.121 | 1.164 | 0.962 | 0.596 | | 0.605 | 0.321 | 1.883 | 0.143 | | 0.652 | 0.320 | 2.035 | 0.103 |  |
|  | HW2×B | - | - | - | - | | 0.487 | 0.314 | 1.552 | 0.266 | | 0.487 | 0.314 | 1.552 | 0.266 |  |
|  | HW1×HW2 | 1.121 | 1.164 | 0.962 | 0.596 | | 0.117 | 0.332 | 0.354 | 0.933 | | 0.164 | 0.331 | 0.496 | 0.873 |  |
| ***Geranium argenteum*** | HW1×B | 2.705 | 0.578 | 4.677 | **<0.001** | | -0.646 | 0.578 | -1.118 | 0.501 | | 0.152 | 0.553 | 0.275 | 0.958 |  |
|  | HW2×B | 1.029 | 0.622 | 1.653 | 0.219 | | -0.639 | 0.505 | -1.264 | 0.414 | | -0.485 | 0.498 | -0.974 | 0.592 |  |
|  | HW1×HW2 | 1.676 | 0.432 | 3.878 | **<0.001** | | -0.007 | 0.545 | -0.013 | 0.999 | | 0.638 | 0.515 | 1.237 | 0.430 |  |
| ***Geum montanum*** | HW1×B | 2.790 | 1.049 | 2.658 | **0.019** | | 2.504 | 0.557 | 4.495 | **<0.001** | | 2.682 | 0.554 | 4.832 | **<0.001** |  |
|  | HW2×B | 1.420 | 1.128 | 1.259 | 0.405 | | 0.816 | 0.329 | 2.477 | **0.033** | | 0.875 | 0.328 | 2.669 | **0.019** |  |
|  | HW1×HW2 | 1.370 | 0.593 | 2.307 | **0.041** | | 1.688 | 0.572 | 2.948 | **0.008** | | 1.806 | 0.569 | 3.170 | **0.004** |  |
| ***Gnaphalium supinum*** | HW1×B | 3.419 | 1.034 | 3.306 | **0.001** | | -0.659 | 0.852 | -0.773 | 0.712 | | 1.683 | 0.483 | 3.480 | **0.001** |  |
|  | HW2×B | 0.000 | 1.422 | 0.000 | 1 | | 1.364 | 0.533 | 2.557 | **0.027** | | 1.181 | 0.501 | 2.358 | **0.046** |  |
|  | HW1×HW2 | 3.419 | 1.034 | 3.306 | **0.001** | | -2.023 | 0.766 | -2.639 | **0.021** | | 0.501 | 0.357 | 1.402 | 0.334 |  |
| ***Homogyne alpina*** | HW1×B | -0.213 | 0.377 | -0.564 | 0.838 | | 2.923 | 0.442 | 6.606 | **<0.001** | | 2.047 | 0.340 | 6.008 | **<0.001** |  |
|  | HW2×B | 0.362 | 0.349 | 1.038 | 0.552 | | 2.146 | 0.435 | 4.930 | **<0.001** | | 1.538 | 0.320 | 4.800 | **<0.001** |  |
|  | HW1×HW2 | -0.575 | 0.362 | -1.588 | 0.250 | | 0.777 | 0.361 | 2.148 | 0.079 | | 0.509 | 0.339 | 1.500 | 0.290 |  |
| ***Hypericum richeri* subsp. *richeri*** | HW1×B | 2.384 | 0.366 | 6.500 | **<0.001** | | -1.556 | 0.540 | -2.877 | **0.010** | | 0.687 | 0.317 | 2.166 | 0.077 |  |
|  | HW2×B | -0.387 | 0.443 | -0.873 | 0.655 | | 0.783 | 0.331 | 2.366 | **0.044** | | 0.632 | 0.315 | 2.006 | 0.110 |  |
|  | HW1×HW2 | 2.772 | 0.403 | 6.878 | **<0.001** | | -2.340 | 0.544 | -4.294 | **<0.001** | | 0.054 | 0.330 | 0.165 | 0.985 |  |
| ***Juncus trifidus* L.** | HW1×B | - | - | - | - | | -0.648 | 0.307 | -2.110 | 0.087 | | -0.648 | 0.307 | -2.110 | 0.087 |  |
|  | HW2×B | - | - | - | - | | -1.145 | 0.311 | -3.681 | **<0.001** | | -1.145 | 0.311 | -3.681 | **<0.001** |  |
|  | HW1×HW2 | - | - | - | - | | 0.496 | 0.302 | 1.643 | 0.227 | | 0.496 | 0.302 | 1.643 | 0.227 |  |
| ***Luzula alpinopilosa* subsp. *alpinopilosa*** | HW1×B | 1.326 | 0.369 | 3.589 | **<0.001** | | -0.708 | 0.436 | -1.624 | 0.235 | | -0.262 | 0.419 | -0.625 | 0.806 |  |
|  | HW2×B | 0.767 | 0.383 | 2.003 | 0.110 | | 0.156 | 0.476 | 0.327 | 0.942 | | 0.325 | 0.468 | 0.694 | 0.766 |  |
|  | HW1×HW2 | 0.559 | 0.321 | 1.738 | 0.189 | | -0.864 | 0.469 | -1.842 | 0.155 | | -0.587 | 0.451 | -1.303 | 0.392 |  |
|  |  |  |  |  |  | |  |  |  |  | |  |  |  |  |  |
| **Species** | **Treatments** | **Germination AUTUMN** | | | | | **Germination SUMMER** | | | | | **Germination FINAL** | | | |  |
|  |  | Estimate | Std.Err | Z-value | P-value | | Estimate | Std.Err | Z-value | P-value | | Estimate | Std.Err | Z-value | P-value |  |
| ***Luzula lutea*** | HW1×B | 0.416 | 0.925 | 0.450 | 0.879 | | -0.013 | 0.439 | -0.030 | 0.999 | | 0.000 | 0.438 | 0.000 | 1 |  |
|  | HW2×B | -0.704 | 1.233 | -0.570 | 0.833 | | -0.066 | 0.431 | -0.155 | 0.986 | | -0.092 | 0.431 | -0.215 | 0.974 |  |
|  | HW1×HW2 | 1.121 | 1.164 | 0.962 | 0.597 | | 0.053 | 0.431 | 0.124 | 0.991 | | 0.092 | 0.431 | 0.215 | 0.974 |  |
| ***Luzula multiflora* subsp. *multiflora*** | HW1×B | 2.286 | 0.409 | 5.578 | **<0.001** | | 1.550 | 0.450 | 3.440 | **0.001** | | 2.193 | 0.426 | 5.145 | **<0.001** |  |
|  | HW2×B | 0.000 | 0.496 | 0.000 | 1 | | -0.351 | 0.317 | -1.106 | 0.504 | | -0.311 | 0.299 | -1.042 | 0.545 |  |
|  | HW1×HW2 | 2.286 | 0.409 | 5.578 | **<0.001** | | 1.901 | 0.453 | 4.198 | **<0.001** | | 2.505 | 0.426 | 5.874 | **<0.001** |  |
| ***Luzula spicata* subsp. *spicata*** | HW1×B | 0.704 | 1.233 | 0.570 | 0.834 | | 1.182 | 0.316 | 3.737 | **<0.001** | | 1.214 | 0.315 | 3.847 | **<0.001** |  |
|  | HW2×B | - | - | - | - | | 1.160 | 0.313 | 3.699 | **<0.001** | | 1.160 | 0.313 | 3.699 | **<0.001** |  |
|  | HW1×HW2 | 0.704 | 1.233 | 0.570 | 0.834 | | 0.021 | 0.327 | 0.066 | 0.997 | | 0.053 | 0.327 | 0.163 | 0.985 |  |
| ***Nardus stricta*** | HW1×B | 1.655 | 1.105 | 1.496 | 0.286 | | 3.627 | 0.626 | 5.788 | **<0.001** | | 3.726 | 0.625 | 5.961 | **<0.001** |  |
|  | HW2×B | - | - | - | - | | 4.268 | 0.631 | 6.757 | **<0.001** | | 4.268 | 0.631 | 6.757 | **<0.001** |  |
|  | HW1×HW2 | 1.655 | 1.105 | 1.496 | 0.286 | | -0.640 | 0.319 | -2.006 | 0.103 | | -0.541 | 0.316 | -1.712 | 0.190 |  |
| ***Phleum alpinum*** | HW1×B | 1.121 | 1.164 | 0.962 | 0.592 | | - | - | - | - | | 1.121 | 1.164 | 0.962 | 0.592 |  |
|  | HW2×B | -0.728 | 0.723 | -1.006 | 0.565 | | - | - | - | - | | -0.728 | 0.723 | -1.006 | 0.565 |  |
|  | HW1×HW2 | 1.849 | 1.090 | 1.695 | 0.200 | | - | - | - | - | | 1.849 | 1.090 | 1.695 | 0.200 |  |
| ***Plantago alpina* subsp. *alpina*** | HW1×B | 2.708 | 0.554 | 4.884 | **<0.001** | | -1.131 | 0.549 | -2.057 | 0.095 | | -0.559 | 0.539 | -1.037 | 0.546 |  |
|  | HW2×B | -0.299 | 0.778 | -0.384 | 0.919 | | 1.159 | 0.831 | 1.394 | 0.337 | | 1.145 | 0.830 | 1.378 | 0.345 |  |
|  | HW1×HW2 | 3.007 | 0.625 | 4.812 | **<0.001** | | -2.290 | 0.796 | -2.874 | **0.010** | | -1.704 | 0.789 | -2.158 | 0.075 |  |
| ***Pulsatilla alpina* subsp. *millefoliata*** | HW1×B | - | - | - | - | | -0.591 | 0.348 | -1.698 | 0.204 | | -0.591 | 0.348 | -1.698 | 0.204 |  |
|  | HW2×B | - | - | - | - | | 0.485 | 0.406 | 1.193 | 0.456 | | 0.485 | 0.406 | 1.193 | 0.456 |  |
|  | HW1×HW2 | - | - | - | - | | -1.076 | 0.384 | -2.799 | **0.014** | | -1.076 | 0.384 | -2.799 | **0.014** |  |
| ***Rumex scutatus* subsp. *scutatus*** | HW1×B | 2.517 | 1.055 | 2.383 | **0.033** | | -1.138 | 0.328 | -3.469 | **0.001** | | -0.878 | 0.318 | -2.756 | **0.016** |  |
|  | HW2×B | 0.000 | 1.422 | 0.000 | 1 | | 0.102 | 0.342 | 0.298 | 0.952 | | 0.116 | 0.342 | 0.341 | 0.937 |  |
|  | HW1×HW2 | 2.517 | 1.055 | 2.383 | **0.033** | | -1.240 | 0.333 | -3.719 | **<0.001** | | -0.994 | 0.323 | -3.073 | **0.005** |  |
| ***Sanguisorba officinalis*** | HW1×B | 1.420 | 1.128 | 1.259 | 0.413 | | -0.418 | 0.316 | -1.323 | 0.382 | | -0.342 | 0.314 | -1.092 | 0.518 |  |
|  | HW2×B | - | - | - | - | | -0.051 | 0.320 | -0.160 | 0.985 | | -0.051 | 0.320 | -0.160 | 0.985 |  |
|  | HW1×HW2 | 1.420 | 1.128 | 1.259 | 0.413 | | -0.367 | 0.314 | -1.166 | 0.473 | | -0.291 | 0.312 | -0.933 | 0.619 |  |
| ***Saxifraga exarata* subsp. *moschata*** | HW1×B | -0.234 | 0.687 | -0.341 | 0.937 | | -0.287 | 1.607 | -0.178 | 0.982 | | -0.299 | 0.778 | -0.384 | 0.921 |  |
|  | HW2×B | -0.740 | 0.631 | -1.173 | 0.468 | | 2.197 | 1.414 | 1.553 | 0.264 | | 0.416 | 0.925 | 0.450 | 0.893 |  |
|  | HW1×HW2 | 0.505 | 0.590 | 0.856 | 0.666 | | -2.484 | 1.384 | -1.794 | 0.170 | | -0.716 | 0.879 | -0.814 | 0.692 |  |
| ***Saxifraga oppositifolia* subsp. *oppositifolia*** | HW1×B | - | - | - | - | | 0.652 | 0.320 | 2.035 | 0.103 | | 0.652 | 0.320 | 2.035 | 0.103 |  |
|  | HW2×B | - | - | - | - | | 0.697 | 0.319 | 2.181 | 0.074 | | 0.697 | 0.319 | 2.181 | 0.074 |  |
|  | HW1×HW2 | - | - | - | - | | -0.045 | 0.302 | -0.151 | 0.987 | | -0.045 | 0.302 | -0.151 | 0.987 |  |
| ***Saxifraga paniculata*** | HW1×B | 0.000 | 1.422 | 0.000 | 1 | | -0.525 | 0.350 | -1.499 | 0.290 | | -0.470 | 0.345 | -1.361 | 0.360 |  |
|  | HW2×B | - | - | - | - | | 0.348 | 0.316 | 1.100 | 0.513 | | 0.348 | 0.316 | 1.100 | 0.513 |  |
|  | HW1×HW2 | 0.000 | 1.422 | 0.000 | 1 | | -0.873 | 0.341 | -2.554 | **0.028** | | -0.819 | 0.337 | -2.426 | **0.040** |  |
| ***Scabiosa lucida*** | HW1×B | 1.095 | 0.334 | 3.276 | **0.002** | | -0.118 | 0.523 | -0.227 | 0.971 | | 0.782 | 0.307 | 2.543 | **0.029** |  |
|  | HW2×B | -0.653 | 0.412 | -1.583 | 0.251 | | 0.926 | 0.407 | 2.272 | 0.058 | | 0.287 | 0.310 | 0.927 | 0.623 |  |
|  | HW1×HW2 | 1.748 | 0.385 | 4.536 | **<0.001** | | -1.045 | 0.473 | -2.205 | 0.069 | | 0.494 | 0.301 | 1.640 | 0.228 |  |
|  |  |  |  |  |  | |  |  |  |  | |  |  |  |  |  |
|  |  |  |  |  |  | |  |  |  |  | |  |  |  |  |  |
| **Species** | **Treatments** | **Germination AUTUMN** | | | | | **Germination SUMMER** | | | | | **Germination FINAL** | | | |  |
|  |  | Estimate | Std.Err | Z-value | P-value | | Estimate | Std.Err | Z-value | P-value | | Estimate | Std.Err | Z-value | P-value |  |
| ***Sempervivum montanum* subsp. *montanum*** | HW1×B | 1.289 | 0.592 | 2.174 | 0.070 | | -0.651 | 0.320 | -2.034 | 0.103 | | -0.420 | 0.306 | -1.369 | 0.356 |  |
|  | HW2×B | -1.420 | 1.128 | -1.259 | 0.405 | | -0.460 | 0.309 | -1.491 | 0.295 | | -0.510 | 0.306 | -1.664 | 0.218 |  |
|  | HW1×HW2 | 2.709 | 1.049 | 2.582 | **0.024** | | -0.190 | 0.311 | -0.611 | 0.814 | | 0.089 | 0.299 | 0.299 | 0.951 |  |
| ***Silene acaulis* subsp. *bryoides*** | HW1×B | 2.572 | 0.629 | 4.084 | **<0.001** | | 0.219 | 0.389 | 0.562 | 0.839 | | 1.084 | 0.323 | 3.351 | **0.002** |  |
|  | HW2×B | 0.299 | 0.778 | 0.384 | 0.919 | | 1.368 | 0.337 | 4.053 | **<0.001** | | 1.306 | 0.323 | 4.033 | **<0.001** |  |
|  | HW1×HW2 | 2.273 | 0.559 | 4.059 | **<0.001** | | -1.149 | 0.361 | -3.176 | **0.004** | | -0.222 | 1.233 | -0.745 | 0.736 |  |
| ***Silene suecica*** | HW1×B | 4.444 | 1.027 | 4.325 | **<0.001** | | -0.640 | 0.371 | -1.721 | 0.196 | | 0.384 | 0.333 | 1.155 | 0.479 |  |
|  | HW2×B | - | - | - | - | | -0.503 | 0.310 | -1.625 | 0.233 | | -0.520 | 0.309 | -1.680 | 0.212 |  |
|  | HW1×HW2 | 4.444 | 1.027 | 4.325 | **<0.001** | | -0.136 | 0.363 | -0.374 | 0.925 | | 0.905 | 0.324 | 2.791 | **0.014** |  |
| ***Solidago virgaurea* subsp. *minuta*** | HW1×B | 3.201 | 0.553 | 5.785 | **<0.001** | | -1.056 | 0.404 | -2.612 | **0.023** | | 0.470 | 0.308 | 1.524 | 0.279 |  |
|  | HW2×B | 0.234 | 0.687 | 0.341 | 0.936 | | -0.210 | 0.306 | -0.686 | 0.769 | | -0.178 | 0.299 | -0.597 | 0.821 |  |
|  | HW1×HW2 | 2.966 | 0.506 | 5.858 | **<0.001** | | -0.845 | 0.404 | -2.089 | 0.090 | | 0.648 | 0.307 | 2.110 | 0.087 |  |
| ***Trifolium alpinum*** | HW1×B | 0.984 | 0.351 | 2.798 | **0.014** | | -0.295 | 0.431 | -0.685 | 0.771 | | 0.454 | 0.302 | 1.500 | 0.290 |  |
|  | HW2×B | 0.145 | 0.381 | 0.380 | 0.923 | | 0.109 | 0.380 | 0.286 | 0.955 | | 0.139 | 0.305 | 0.457 | 0.891 |  |
|  | HW1×HW2 | 0.839 | 0.342 | 2.451 | **0.037** | | -0.404 | 0.429 | -0.943 | 0.611 | | 0.314 | 0.300 | 1.047 | 0.546 |  |
| ***Vaccinium uliginosum* subsp. *microphyllum*** | HW1×B | 2.796 | 1.046 | 2.671 | **0.019** | | -0.659 | 0.359 | -1.834 | 0.158 | | -0.430 | 0.353 | -1.219 | 0.441 |  |
|  | HW2×B | 0.704 | 1.233 | 0.570 | 0.830 | | -0.014 | 0.373 | -0.037 | 0.999 | | -0.000 | 0.372 | -0.000 | 1 |  |
|  | HW1×HW2 | 2.092 | 0.771 | 2.710 | **0.017** | | -0.645 | 0.359 | -1.793 | 0.171 | | -0.430 | 0.353 | -1.219 | 0.441 |  |
| ***Vaccinium myrtillus*** | HW1×B | 4.533 | 1.027 | 4.411 | **<0.001** | | -0.590 | 0.372 | -1.584 | 0.250 | | 0.677 | 0.314 | 2.152 | 0.079 |  |
|  | HW2×B | - | - | - | - | | 0.905 | 0.324 | 2.791 | **0.014** | | 0.905 | 0.324 | 2.791 | **0.014** |  |
|  | HW1×HW2 | 4.533 | 1.027 | 4.411 | **<0.001** | | -1.496 | 0.392 | -3.810 | **<0.001** | | -0.227 | 0.338 | -0.673 | 0.778 |  |
